# OSCAR4: a flexible architecture for chemical text-mining

**DOI:** 10.1186/1758-2946-3-41

**Published:** 2011-10-14

**Authors:** David M Jessop, Sam E Adams, Egon L Willighagen, Lezan Hawizy, Peter Murray-Rust

**Affiliations:** 1 Unilever Centre for Molecular Science Informatics, Department of Chemistry, Lensfield Road, Cambridge CB2 1EW, UK

## Abstract

The Open-Source Chemistry Analysis Routines (OSCAR) software, a toolkit for the recognition of named entities and data in chemistry publications, has been developed since 2002. Recent work has resulted in the separation of the core OSCAR functionality and its release as the OSCAR4 library. This library features a modular API (based on reduction of surface coupling) that permits client programmers to easily incorporate it into external applications. OSCAR4 offers a domain-independent architecture upon which chemistry specific text-mining tools can be built, and its development and usage are discussed.

## Introduction

*In keeping with the historical and methodological aspects of this special issue, we recount the history and motivation of OSCAR*.

A large amount of factual data in chemistry and neighbouring disciplines is published in the form of text and components within text rather than as structured semantic information. If we can discover and extract this information, the textual literature becomes an enormous additional chemical resource. As an example, we estimate that about 10 million chemical syntheses per year are published in the public literature (articles, patents, theses) and the conventional method is a natural language narrative (most commonly in English). It is extremely tedious and error-prone to extract information from this narrative manually, and for this reason many chemical abstracting services limit their scope and also frequently lag behind the current publication list.

The discipline of text-mining has now reached a state where much natural language in textual form can be analysed rapidly and with high precision and recall. Methodologies applied to the problem of chemical named entity recognition include dictionary- and rule-based methods, as well as machine learning and hybrid approaches [1-11]. We have been working in this area for approximately 10 years and the OSCAR4 software, together with OPSIN (the Open Parser for Systematic IUPAC Nomenclature) [12,13] and ChemicalTagger [14,15], represent the public state-of-the-art in chemical text analysis and extraction.

The OSCAR (Open-Source Chemistry Analysis Routines) software has been developed over a period of years and a number of projects. Between 2002 and 2004, sponsors including the Royal Society of Chemistry (RSC), Nature and the International Union of Crystallography (IUCr) supported a number of summer studentships. These projects were focused on the development of software with limited capacity for the automated interpretation of chemical documents, and resulted in two main software components-the Experimental Data Checker [16,17] and OSCAR2.

**Figure 1:**
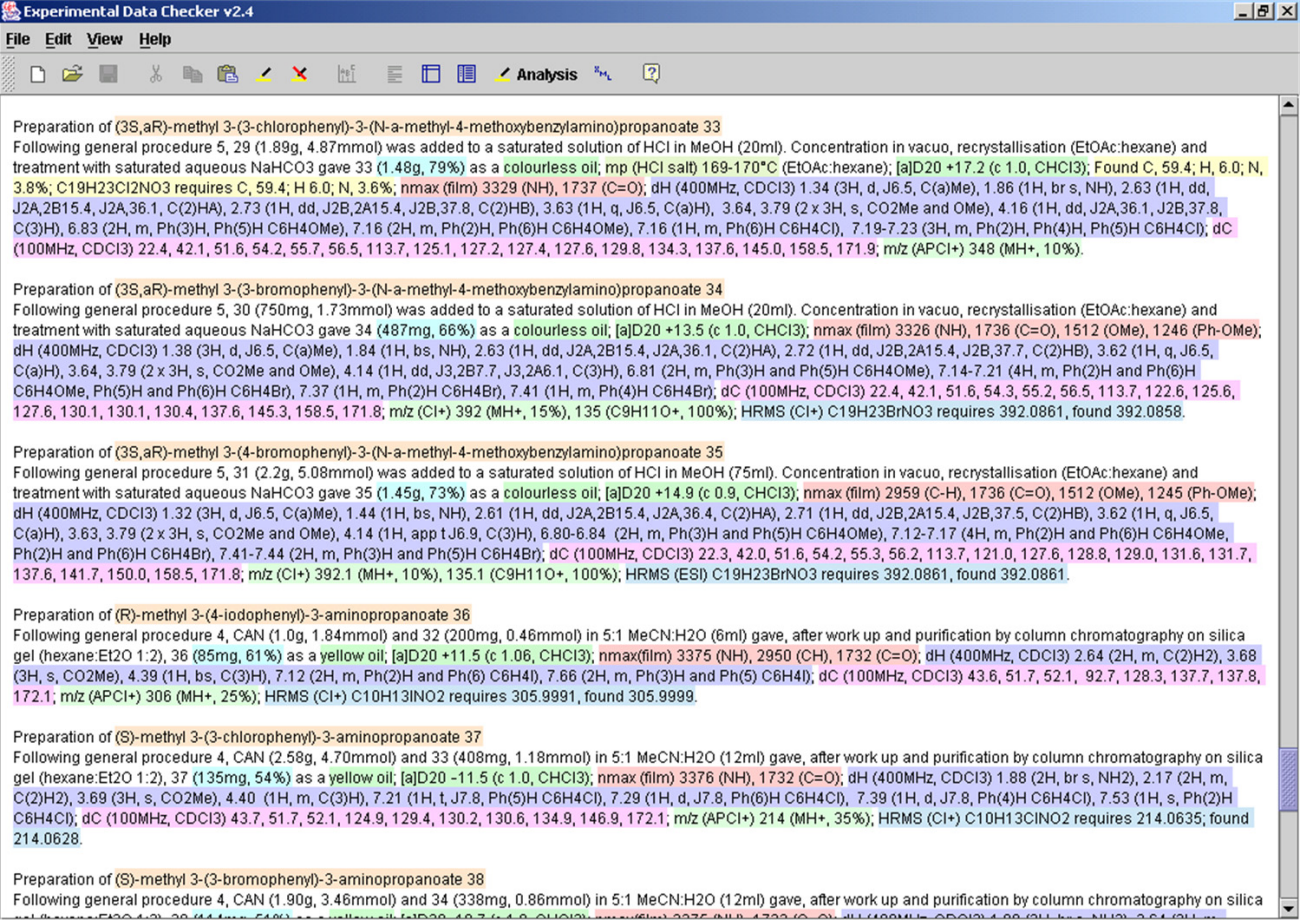
**A screenshot of the Experimental Data Checker (OSCAR-Data) showing identification and markup of plain text experimental data**. The initial application of OSCAR was to parse the highly stylised data used to report spectra and other analytical proofs of synthesis. This functionality is very widely-used (pers. comm. from RSC staff) and has been re-integrated into OSCAR4 rather than being a separate application.

The Experimental Data Checker was conceived as a tool to be used as part of the RSC's publication process. The tool is capable of recognising sections of reported experimental data within plain text input using regular expressions to match the highly-stylised and journal-mandated formats in which they are reported in the literature (as shown in Figure 1). Once this information has been identified and interpreted, the tool performs elementary checks on the characterisation data (spectra, analytical) where molecular structures are reported, and attempts to ensure that the data does not conflict with the structure.

The Experimental Data Checker application relied upon a core library of analysis routines, and it was this library that was the first to bear the name OSCAR. Further development of this library in the summer of 2004 resulted in OSCAR2, which used XML formatting to represent the document undergoing processing, and applied XML annotations to the document to indicate recognised sections of text. OSCAR2 implemented a naïve Bayesian system based on *n*-grams and a simple grammar in order to identify chemical names within a text. These improvements were later extended as part of the OSCAR3 project.

In 2005, the EPSRC awarded a grant ("Sciborg") to develop natural language processing (NLP) tools for chemistry and science. The chemistry component of this project focused on the development of the OSCAR2 methodology and resulted in the creation of OSCAR3 [18]. OSCAR3 focuses on the recognition of and, where appropriate, the resolution of connection tables for chemical named entities. OSCAR3 employs a naïve Bayesian model to identify "chemical" tokens in text and offers a choice of two methods for the identification of multi-token named entities. The first of these, the PatternRecogniser, uses predetermined regular-expression style heuristics while the second, the MEMMRecogniser [19], employs machine learning in the form of a Maximum Entropy Markov Model (MEMM). OSCAR3 uses these methods to identify four classes of named entity (Chemical, Reaction, Chemical Adjective and Enzyme) as well as dictionary lookup to identify a pre-determined set of ontology terms and a discrete finite automaton based method to identify chemical prefixes.

**Figure 2:**
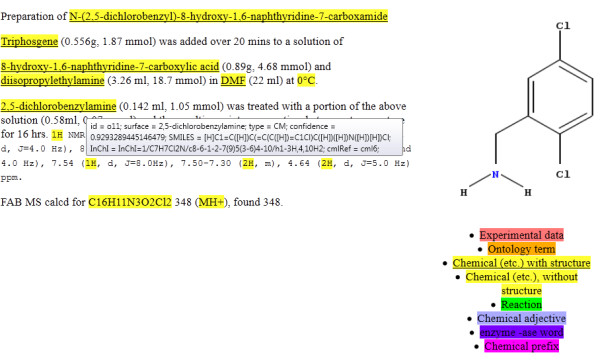
**OSCAR3 markup displaying recognised chemical entities (CM)**. A mouse-over action on an annotated term displays the associated metadata, in this case for 2,5-dichlorobenzylamine, and displays an image representing the structure generated by the Chemistry Development Kit (CDK) [35-37] (right). OSCAR3 concentrated on the identification and interpretation of chemical entities in text (named entity recognition, NER). The primary purpose was to identify and extract the following types of object: chemicals (CM), ontology terms (ONT; looked-up from ChEBI [38-40], FIX [41] and REX [42]*etc*.), reactions (RN; as identified by linguistic constructs, *e.g*. "methylated"), chemical adjectives (CJ) mainly formed from chemical nouns), enzymes (ASE) and chemical prefixes (CPR), highlighted in different colours. These concepts are maintained in OSCAR4.

In order to convert chemical names to connection tables (Figure 2), OSCAR3 uses dictionary-based methods and, where this is not successful, OPSIN. Early versions of OSCAR directly included the OPSIN code, but this was later re-factored into a separate library.

By 2008, OSCAR was in common use in many laboratories for the identification and extraction of chemical terms (chemical named entities) in a variety of texts. Our original metrics [18] showed that the precision and recall were domain-dependent and varied considerably with the purpose and style of chemical texts. Feedback from users was informal but it was clear that they were modifying OSCAR for their particular purposes both in vocabulary and recognition methods. As a result we embarked on a major re-factoring program in order to robustify the OSCAR software and simplify the API, and this paper describes the results.

### Historical Funding and Collaboration

It is very difficult to get funding for software engineering projects, especially when apparently little changes on the surface. We are grateful to the following bodies for their funding and interest:

1. OMII-UK. This organisation existed to support and robustify the products of the UK eScience program. Many of these were middleware products but OSCAR was seen by the UK eScience community as an example of a widely-deployable component that could be used in a modern manner in many branches of science. The OMII-UK project carried out an initial scoping and re-factoring of the OSCAR3 source.

2. The OSCAR-ChEBI project. This was a competitive funding resource for eScience products and we worked with the European Bioinformatics Institute (EBI) to develop OSCAR as an appropriate tool for the extraction and verification of chemistry in the ChEBI ontology.

3. CheTA. This was a JISC-funded project led by our group in conjunction with the National Centre for Text Mining (NaCTeM) to evaluate the relative merits of human annotation and machine annotation of documents. Part of this project involved OSCAR running under the UIMA [20]/U-Compare [21,22] framework and required a re-factoring [23].

As a result of these projects, which probably amounted to two person-years of effort in the re-factoring, OSCAR4 has now been released in a usable form.

### Limitation of OSCAR3 and design goals for OSCAR4

OSCAR3 is a powerful tool for chemical natural language processing, but early attempts to develop software using it as a library rather than as a standalone application-the ChemicalTagger [14] and PatentEye [24,25] projects-exposed weaknesses in the code in this regard. The architecture of the software was built around the principle that the software would be running as a server on the user's local machine. In order to function correctly, it required a properly configured workspace. Many key components were implemented as mutable singletons (static objects), compromising the thread-safety of the application and meaning that safe reconfiguration of a workflow required a complete shutdown and restart of the Java virtual machine (JVM). Furthermore, the implementations of the various OSCAR components required that a document be formatted in SciXML as it underwent processing. Consequently, the use of OSCAR3 by a client programmer to build secondary applications was unintuitive, and the distribution and successful use of such applications was found, as part of the Green Chain Reaction, to require an unacceptably high level of support.

Early attempts to resolve these problems [23] involved the extraction of the OSCAR3 tokeniser, MEMMRecogniser and PatternRecogniser components from the main OSCAR3 codebase and their conversion into modules suitable for use in the popular text-mining framework U-Compare. This work allowed the use of OSCAR as part of a drag-and-drop workflow, but not its direct integration into another application. Consequently, a comprehensive overhaul of the OSCAR3 code began in autumn 2010 with the aim of producing a well-engineered, simple, modularised version of OSCAR that retained the core OSCAR3 functionality and could be easily integrated into external applications. This most recent development has been designated OSCAR4 and is discussed in the remainder of this paper.

The development of OSCAR4 sought to address a number of specific issues. These are summarised below (and in Appendix A) and subsequently discussed in greater detail.

1. To produce an OSCAR library with a simple API, suitable for use by client programmers who may not be familiar with the internal workings of OSCAR. Consequently, while it is desirable for users to be able to customise the behaviour of OSCAR in a number of ways, initialisation of OSCAR components must by default produce configurations that "just work"-the 'convention over configuration' paradigm (Appendix B).

2. In order to run, OSCAR3 required the existence of a properly configured workspace-a directory on the executing machine that contains the OSCAR chemical name dictionary, the InChI [26,27] binary file and a properties file along with subdirectories intended to contain further resource files. When OSCAR3 is first run this workspace is automatically created, and when OSCAR3 is used as a library the workspace is automatically created in the working directory. This behaviour was deemed undesirable, unnecessary and found to be a cause of difficulties in producing distributable OSCAR-dependent software. Consequently, the removal of the requirement for a workspace was considered a high priority of the OSCAR4 project.

3. Much of the OSCAR3 code required that a document undergoing processing is formatted in SciXML. Though converters are provided to transform HTML into plain text and plain text into SciXML, the requirement to perform this transformation is frustrating to the client programmer in that it prevents him from working directly with plain text or with a custom XML format which may very well be the native format of a document that he wishes to process. Consequently, the removal of this SciXML dependence was considered important.

4. In addition to its core functionality-the recognition and interpretation of chemical named entities-OSCAR3 included a wide range of secondary functions including the OSCAR3 server. This server runs on the local machine and provides an interactive demonstration of the capacity of OSCAR3 for text processing as well as a number of other utilities including the capacity to manually annotate a text from within a browser window, a servlet for the interconversion and depiction of chemical names and formats and an experimental Hearst pattern [28] based system for the extraction of chemical relations from text. The OSCAR3 codebase had the resemblance of a 'treasure trove' which made code maintenance a more complex task than necessary. The separation of a library containing the core OSCAR functionality from these secondary functions was therefore considered desirable.

5. Much of the architecture of OSCAR3 lacked clear definition. Excessive use is made of mutable singletons which, while aiding performance by eliminating the need for re-initialisation of components, allows for complex interactions in the code, making it difficult to understand, debug and re-factor. This problem was compounded by the manner in which program logic is partially controlled by a properties object backed by a serialised file. Some of the property values can be modified at runtime while others, once accessed by the objects that rely upon them, are duplicated in memory and cannot be further changed. Attempts to resolve these complex interactions can have unintended consequences since the unit test coverage in OSCAR3 is sparse. Consequently, the improvement of the architecture of the OSCAR software was considered a vital part of the OSCAR4 project.

6. It has been known for some time that the speed of OSCAR3 operation could be improved by introducing certain optimisations into the code. Using the YourKit Java profiler [29], a number of performance blackspots were identified and subsequently eliminated. This work was started after the final version of OSCAR3 (OSCAR3 alpha 5 [30]) and continued as part of the OSCAR4 project.

### Library as a design

**Figure 3:**
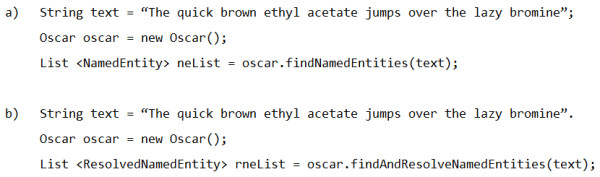
**Java code using the OSCAR4 API to a) identify chemical named entities (CNEs) in a block of text and b) identify CNEs and resolve their connection tables where possible**.

OSCAR4 has been deliberately written as a Java library, rather than an application or service. Consequently, the decoupling of the core OSCAR functionality from applications that use this functionality has been achieved. The usage of the library has been simplified as much as possible with the introduction of the Oscar API object-a class intended to wrap the functionality of the wider library and provide default implementations of the various components. As a result, OSCAR4 can be called from external software, as shown in the examples in Figure 3.

**Figure 4:**
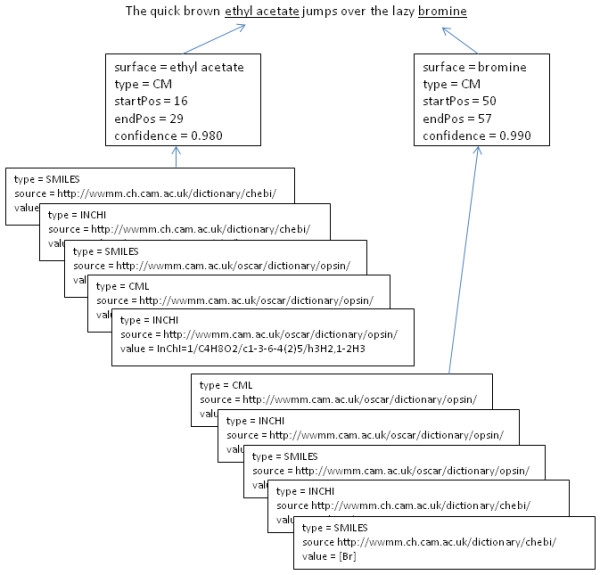
**Graphic representing the structure of the OSCAR4 API output object**. Named entities reference their position in the input text, the confidence in their identification and resolved structures in various formats (SMILES [43,44], InChI, CML [45]*etc*.).

In the first of the examples in Figure 3, OSCAR4 is used to detect named entities in an input string, returning a List of NamedEntity objects. In the second, it is used to both detect named entities and, where these named entities correspond to chemical names, to resolve these names to chemical structures-returning a List of ResolvedNamedEntity objects. The ResolvedNamedEntity class links a NamedEntity to a list of chemical structures in a number of formats-SMILES, InChI and CML-while the NamedEntity class stores such information as the surface (raw text) and type (*e.g*. compound or reaction) of the named entity and the indices that define its position within the source text. The outputs of these examples are illustrated in Figure 4.

**Figure 5:**
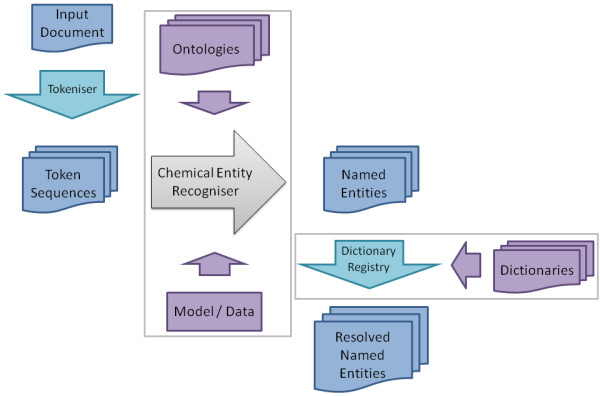
**Workflow of the OSCAR4 API object**.

The examples above show how OSCAR4 can be used without the need for any understanding of the underlying technology or implementations. An overview of the workflow managed by the Oscar API object is shown in Figure 5.

The input is first passed to the Tokeniser to produce a list of TokenSequence objects, each of which roughly corresponds to a paragraph of text and contains a list of Token objects. The Token represents a string of characters that mostly correspond to words but also to punctuation or other discrete units of text *e.g*. "C_2_H_6_O" or "42". In NLP tools, tokenisation commonly occurs at whitespace or punctuation boundaries, however due to the form of some of the domain-specific entities found in chemical texts such as "C-H" a custom Tokeniser is used. The TokenSequences are then passed to a ChemicalEntityRecogniser-an interface for a class capable of identifying a list of NamedEntities, which are subsequently passed to the ChemNameDictRegistry to create a list of ResolvedNamedEntities if required.

This workflow can be customised by the user, who can use the set() methods of the Oscar class to replace the components of the default configuration with suitable customised or custom-built alternatives. Specifically, the user can select which implementation of ChemicalEntityRecogniser to use or can specify which set of ontology terms are to be recognised and which model the default ChemicalEntityRecogniser should use, and which dictionary registry, *i.e*. set of chemical name dictionaries, to use for name to structure resolution. In addition to this, the public APIs of the individual components can be used to assume a greater degree of control over the execution of the workflow.

OSCAR4 provides three implementations of the ChemicalEntityRecogniser. The first, the RegexRecogniser, finds terms that match a given regular expression and is intended to find serial numbers corresponding to compounds *e.g*. "NSC-2648". The others, the PatternRecogniser and the MEMMRecogniser, use more complex strategies to identify chemical named entities and feature subcomponents that can be customised by the user to produce the desired behaviour.

**Figure 6:**
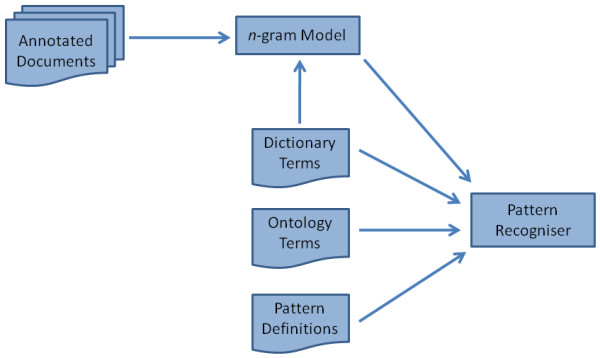
**PatternRecogniser architecture**.

The architecture of the PatternRecogniser is shown in Figure 6. A list of "chemical" words is drawn from an internal dictionary composed mostly of words derived from the ChEBI database and from a corpus of manually-annotated documents, while a list of "non-chemical" words is determined by removing those words that occur in the chemical word list from a standard English dictionary. These lists are used to build an *n*-gram model which is used by a naïve Bayesian classifier to determine whether novel tokens are "chemical" or "non-chemical". Multi-token named entities, *e.g*. "ethyl acetate", that occur within the input text are then identified by regex-style matching of chemical tokens to a set of pre-specified pattern definitions such as "*yl *ate".

**Figure 7:**
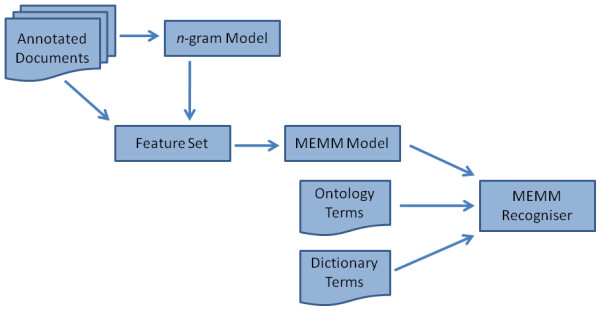
**MEMMRecogniser architecture**.

The architecture of the MEMMRecogniser is shown in Figure 7, in which chemical named entities are identified using a Maximum Entropy Markov Model (MEMM). The feature set that is generated for each token includes features that describe the token in question, such as the *n*-grams that describe it and the probability that it is chemical as predicted by the *n*-gram model as previously, as well as contextual features that describe its neighbouring tokens. Using these features, the MEMM model assigns a chemical token as being either the first token in a named entity or a subsequent token in a named entity. Given these assignments, multi-token named entities can be constructed. Novel MEMM models can be built from a corpus of hand-annotated documents by the user, and OSCAR4 is supplied with two pre-generated models. One of these models was built from a set of papers from RSC journals [31], while the other was built from a set of abstracts retrieved from PubMed [19].

### Architecture and tests

The OSCAR4 library has been separated into a number of modules with each performing a defined role in the operation of the OSCAR code, such as the tokenisation of text or the provision of chemical name dictionaries. This allows client programmers to use as much or as little of OSCAR in their applications as required, without the need to unnecessarily pull in a large, comprehensive, single JAR. The process of creating the sub-projects had the additional advantage of highlighting the ways in which the separate components interact. During this process, the readability of the OSCAR code was improved by imposing a number of the idioms of 'clean code', and the reliability of the code was improved by the creation of appropriate unit and regression tests. At the time of writing, OSCAR4 has nearly 500 tests. As a result, the OSCAR4 code is far more robust than OSCAR3, so a developer can work both with and on the core OSCAR code with a far greater degree of confidence.

The mutable singletons that were commonplace in OSCAR3 have been largely removed. Instead, when setting up custom workflows, a user has the choice of either calling the getDefaultInstance() method or the default constructor as appropriate-each of which returns a preconfigured instance of the class-or using the custom constructor which uses dependency injection to supply the OSCAR components upon which the class depends. For example, the OntologyTerms class represents a set of ontology terms and their corresponding ontology IDs. The following two methods of obtaining an OntologyTerms object are available:

OntologyTerms.getDefaultInstance();

new OntologyTerms(ListMultimap < String, String > terms);

The first method returns the default OSCAR4 OntologyTerms object, which contains an amalgamation of the terms from the ChEBI, FIX and REX ontologies while the second supplies a multimap of ontology terms to IDs. The use of this design pattern throughout the codebase permits, but by no means requires, a user to assume a high degree of control over the functioning of OSCAR.

The use of the properties file and object to control elements of the program execution has been removed. Instead, the required information is either specified as part of a constructor's signature or using a set() method on the object in question. This improves the thread-safety of OSCAR, particularly in a multiuser environment, and contributes to its usability since a user can now trivially see what features may be customised from the outline of the class as opposed to needing to know which and how properties are used by which components.

### Input and Output Formats

As previously discussed, OSCAR3 required that input documents be converted into SciXML before processing can occur, using the document formatting as a base against which annotations for identified named entities can be referenced-whether as inline or standoff annotations. XML input turned out to be overly complex as NLP tools require "flat" relatively sequential tokens. The XML markup adds little useful context. OSCAR4 removes this requirement by operating on plain text and producing NamedEntity and DataAnnotation objects to represent recognised sections of text and does not currently produce serialised output, though some support for the serialisation of annotations into XML documents is planned for future releases. It should be realised, however, that there is no single, fool-proof approach to this problem. Different XML schema may use different methods to indicate where in the document section breaks and even text content occur, while it cannot be guaranteed that well-formed inline annotations can be generated for a given input document. Client programmers are therefore recommended to consume NamedEntity objects directly rather than rely upon serialised output, though it is realised that users are likely to want to be able to create serialised, marked-up copies of their documents as well.

### Non-core functionality

Non-critical code (particularly downstream applications) has been removed from the OSCAR4 codebase to reflect the philosophy that OSCAR4 should act as a library. While some minor supporting code remains, such as that required for generation of key resource files, the majority has been removed entirely as it is envisaged that much of the former functionality could be better implemented by developers with specific use cases.

**Figure 8:**
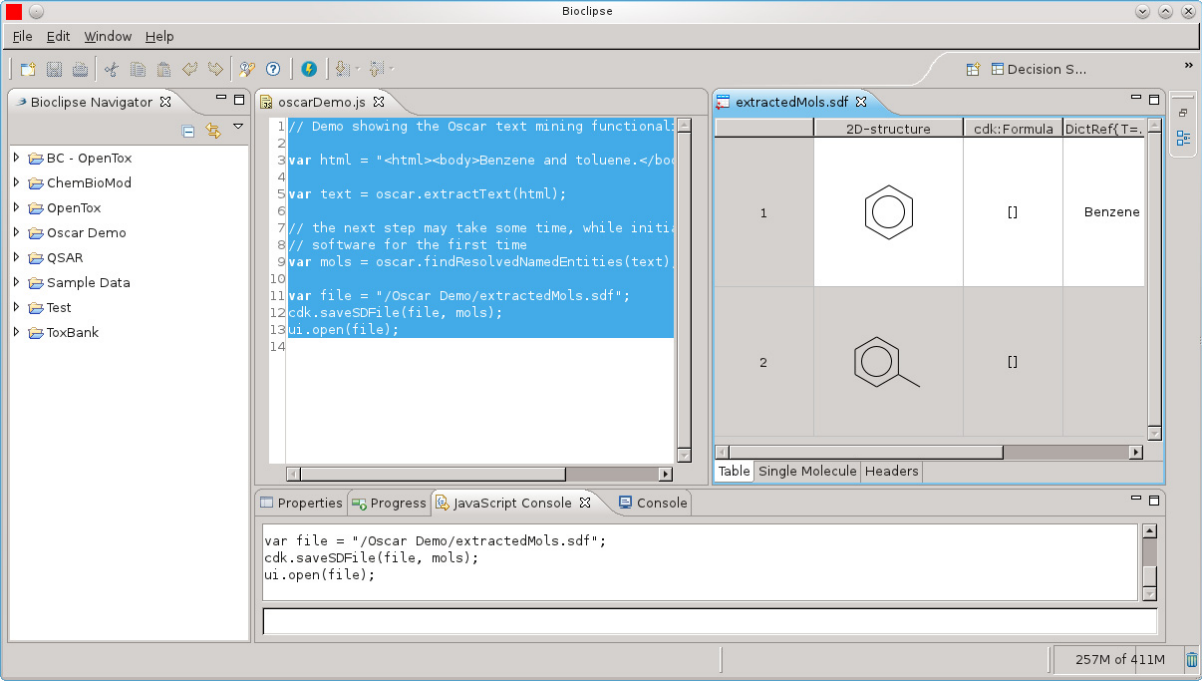
**OSCAR4 run within Bioclipse's scripting interface (centre pane) identifying named entities in a block of text and saving the connection tables to file (extractedMols.sdf) for viewing (right pane)**.

A number of useful non-core functions are provided in dependent libraries developed at the Unilever Centre in Cambridge. Specifically, subsidiary modules exist to provide the capacity to run OSCAR4 from the command-line, as part of UIMA or Taverna [32] workflows and from the Bioclipse [33] scripting interface, as shown in Figure 8.

### Performance

A number of modifications were introduced to the OSCAR code with the aim of reducing the time required to process documents. Performance hotspots were identified using the YourKit Java profiler and where possible eliminated. Some such improvements focused on the time taken to initialise the various OSCAR components, such as supplying a pre-calculated, serialised copy of the *n*-gram models used for named entity recognition rather than regenerating them each time OSCAR is loaded. Others improved the speed at which OSCAR can process a document by optimising extremely tight loops in the code, such as eliminating unnecessary string declaration while calculating *n*-gram features and avoiding recompilation of regular expressions. Further improvements were made *ad hoc*, as the OSCAR4 developers encountered obvious bottlenecks while working on the code.

In order to quantify the improvement in speed of operation, the time taken by both OSCAR4 version 4.0.1 and OSCAR3 alpha 5 to perform two tasks was measured. The first task measured the time taken to initialise the software to the point that it was ready to begin the task of finding named entities in text; the second task aimed to measure the speed at which the software could process bulk text and consisted of processing the full text of the 68 patents published by the European Patent Office in the week of 2009-05-06-a total of 11468 paragraphs of text. All the tasks were run on a desktop computer equipped with an Intel Pentium 4 (3.00 GHz) CPU and 1 GB of RAM, purchased *c*. 2005, running openSUSE 11.1 and using the Java 1.6.0_22 32-bit virtual machine with a maximum heap size of 512 MB. The results are summarised in Table 1 and Table 2.

**Table 1:** Results of the initialisation task

Software version	OSCAR4 4.0.1	OSCAR3 alpha 5	OSCAR4 4.0.1	OSCAR3 alpha 5
**Recogniser**	**MEMMRecogniser**	**MEMMRecogniser**	**PatternRecogniser**	**PatternRecogniser**

Mean time (s)	14.4	17.3	19.7	24.6

Standard deviation (ms)	40.8	40.0	72.6	88.6

**Table 2:** Results of the bulk processing task

Software version	OSCAR4 4.0.1	OSCAR3 alpha 5	OSCAR4 4.0.1	OSCAR3 alpha 5
**Recogniser**	**MEMMRecogniser**	**MEMMRecogniser**	**PatternRecogniser**	**PatternRecogniser**

Mean time (s)	446	541	150	276

Standard deviation (s)	1.85	1.14	0.556	1.53

From these data, it can be seen that OSCAR4 performs significantly faster than OSCAR3. Initialisation times for the MEMMRecogniser and PatternRecogniser have been reduced by 17% and 20% respectively, while bulk processing times have been reduced by 18% and 46% respectively. The OSCAR4 MEMMRecogniser and PatternRecogniser processed approximately 26 and 76 paragraphs per second respectively, demonstrating that bulk processing of text is achievable on an acceptable timescale on desktop computers.

### Deployment

OSCAR4 has generated significant interest in the community, and has been the subject of two meetings at the Unilever Centre for Molecular Science Informatics in Cambridge. The talks from the second of these are available to view online [34]. To our knowledge, the software is in use at the National Centre for Text Mining (NaCTeM), the European Bioinformatics Institute (EBI) and the European Patent Office (EPO) as well as various pharmaceutical companies.

We are aware of successful and straightforward integrations into the Bioclipse and Taverna frameworks, and believe that this is similarly straightforward for other Java environments. We were also pleased to see that at the recent MIOSS meeting at the EBI, OSCAR and OPSIN had been integrated into the .NET environment. For example, OPSIN was demonstrated as running within the JVM in Microsoft Excel, which is acceptable to commercial organisations as the JVM is of proven security.

### Future Prospects

This is a useful opportunity to reflect on the high cost of producing robust, re-usable software. OSCAR3, and OPSIN, were produced as a continuing activity by a mixture of summer students, PhDs and PDRAs and, until *ca*. 2009, evolved rather than having a top-down software design. When the project became valuable to the world, it was a clear indication that re-factoring was going to be essential, and it is important to realise the necessary but high cost of doing this. In times of lean funding, it will become increasingly difficult to obtain this type of support, and therefore it is always tempting to transfer academic code to commercial entities which can raise revenue.

The downside of this is that we know of very few commercial codes, and certainly none in chemical text analysis, that provide public metrics let alone expose the architecture on which the program is based. Text-mining as an academic subject requires metrics and increasingly requires Openness of the components of the system, as we have done in OSCAR and OPSIN. We are investigating continuing business models where we can continue to re-factor and improve the product while not closing the code and therefore reducing scientific credibility and innovation.

Very recently we have been exploring the use of OSCAR for areas other than organic and biological chemistry. Because OSCAR can be customised by different dictionaries, we have been able to adapt it to process reports of atmospheric chemistry and, more generally, atmospheric science. In conjunction with the European Geosciences Union (EGU, which publishes Open Access papers), we have analysed abstracts and full text for chemical entities and related numerical quantities (*e.g*. amounts, conditions *etc*.) This has led to a design where the domain-independent parts of OSCAR4 can be applied to many physical sciences with bespoke dictionaries and ChemicalTagger rules. We have submitted grants in both the biosciences ("OSCAR-BIO") and physical sciences ("OSCAR-PHYS"). As part of this work, we will be actively addressing generic tools for metrics and training.

## Competing interests

The authors declare that they have no competing interests.

## Authors' contributions

DMJ wrote the manuscript and was lead developer in the OSCAR4 re-factoring.

SEA was the architect and project manager for the OSCAR4 re-factoring and was involved in the original Experimental Data Checker project.

ELW contributed to the OSCAR4 re-factoring and investigated its use in Bioclipse.

LH contributed to the OSCAR4 re-factoring and was involved in the CheTA project.

PMR had the overall vision for, and was involved in, all stages of the various OSCAR projects, and wrote the manuscript.

All authors have read and approved the final version.

## Appendixes

### Appendix A: Additional OSCAR4 resources

The source code, mailing list, tutorials, documentation and support are available at

https://bitbucket.org/wwmm/oscar4/wiki/Home

This page also includes instructions for accessing pre-compiled JAR files from the Unilever Centre's Maven repository.

The source code used to measure OSCAR performance is available at https://bitbucket.org/dmj30/oscar-performance

The OSCAR4 Javadoc is available at http://apidoc.ch.cam.ac.uk/oscar4-4.0.1

### Appendix B: Building on the OSCAR4 API

The core methods are given in each case. In some cases it will be valuable to extract further information recursively from the results.

a) Searching a given text for Named Entities. These can then be displayed, computed *etc*.

Oscar **oscar **= new Oscar();

List < NamedEntity >**namedEntities**

= **oscar**.**findNamedEntities**(**text**);

b) Where the named entity can be resolved to a chemical structure, extract it:

Oscar **oscar **= newOscar();

List < ResolvedNamedEntity >**entities**

= **oscar**.**findAndResolveNamedEntities**(**s**);

for (ResolvedNamedEntity **entity **: **entities**) {

ChemicalStructure **structure **= **entity**.**getFirstChemicalStructure**http://(FormatType.INCHI));

...

}

c) Find only those entities which are resolvable to structures (*e.g*. "benzene" but not " the methyl ester":

Oscar **oscar **= newOscar();

List < ResolvedNamedEntity >**entities**

= **oscar.findResolvableEntities(s)**;

d) Tailor the system to use different recognizers and dictionaries:

ChemicalEntityRecogniser **myRecogniser **= newPatternRecogniser()

Oscar **oscar **= newOscar();

**oscar.setRecogniser(myRecogniser)**;

**oscar.setDictionaryRegistry(myDictionaryRegistry)**;

List < ResolvedNamedEntity >**entities **= **oscar.findResolvableEntities(s)**;
